# Rosiglitzone Suppresses Angiotensin II-Induced Production of KLF5 and Cell Proliferation in Rat Vascular Smooth Muscle Cells

**DOI:** 10.1371/journal.pone.0123724

**Published:** 2015-04-14

**Authors:** Dengfeng Gao, Guanghua Hao, Zhe Meng, Ning Ning, Guang Yang, Zhongwei Liu, Xin Dong, Xiaolin Niu

**Affiliations:** 1 Department of Cardiology, The Second Affiliated Hospital, Xi’an Jiaotong University, Xi’an, Shaanxi 710004, P.R. China; 2 Department of Nuclear medicine, The Second Affiliated Hospital, Xi’an Jiaotong University, Xi’an, Shaanxi 710004, P.R. China; Chang Gung University, TAIWAN

## Abstract

Krüppel-like factor (KLF) 5, which initiates vascular smooth muscle cell (VSMC) proliferation, also participates in Angiotensin (Ang) II-induced vascular remodeling. The protective effect of rosiglitazone on vascular remodeling may be due to their impact on VSMC proliferation. However, the underlying mechanisms involved remain unclear. This study was designed to investigate whether the antiproliferation effects of rosiglitazone are mediated by regulating Ang II/KLF5 response. We found that, in aortas of Ang II-infused rats, vascular remodeling and KLF5 expression were markedly increased, and its target gene cyclin D1 was overexpressed. Co-treatment with rosiglitazone diminished these changes. In growth-arrested VSMCs, PPAR-γ agonists (rosiglitazone and 15d-PGJ2) dose-dependently inhibited Ang II-induced cell proliferation and expression of KLF5 and cyclin D1. Moreover, these effects were attenuated by the PPAR-γ antagonists GW9662, bisphenol A diglycidyl ether and PPAR-γ specific siRNA. Furthermore, rosiglitazone inhibited Ang II-induced phosphorylation of protein kinase C (PKC) ζ and extracellular signal-regulated kinase (ERK) 1/2 and activation of early growth response protein (Egr). In conclusion, in Ang II-stimulated VSMCs, rosiglitazone might have an antiproliferative effect through mechanisms that include reducing KLF5 expression, and a crosstalk between PPAR-γ and PKCζ/ERK_1/2_/Egr may be involved in. These findings not only provide a previously unrecognized mechanism by which PPAR-γ agonists inhibit VSMC proliferation, but also document a novel evidence for the beneficial vascular effect of PPAR-γ activation.

## Introduction

Vascular remodeling is closely involved in the progression of atherosclerosis and restenosis, and is also present in hypertension- and diabetes-induced vascular complications[1]. Proliferation of vascular smooth muscle cells (VSMCs) is a major cellular event of this process [2]. Accumulating evidence shows that Ang II is also capable of inducing VSMC proliferation and has emerged as a major driving force of vascular remodeling[2].

Krüppel-like factor (KLF) 5, also known as basic transcription element binding protein 2, belongs to the Krüppel-like transcription factor family. Members of this family contain 3 Krüppel-like C2H2-type zinc (Zn)-finger domains, recognize GC boxes, and have diverse functions in cell proliferation, differentiation, and embryonic development[3]. In the cardiovascular system, KLF5 is abundantly expressed in developing blood vessels, but is downregulated in adult vessels [4]. However, its expression is strongly upregulated in activated VSMCs within vascular lesions [5,6]. In addition, *in vitro* analyses of VSMCs show that KLF5 activates many types of genes such as cyclin D1, inducible nitrc oxide synthase, plasminogen activator inhibitor 1, transforming growth factor β and vascular endothelial growth factor receptors, which are known to be induced during cardiovascular remodeling[7,8]. Moreover, we and other researchers have proved that KLF5 is a target for Ang II signaling and an essential regulator of cell proliferation in VSMCs [9–12]. Therefore, KLF5 might provide an important link between Ang II, cell proliferation and vascular remodeling [11].

Thiazolidinediones (TZDs) such as rosiglitazone are high-affinity ligands for peroxisome proliferator-activated receptor γ (PPAR-γ), a transcription factor of the nuclease hormone receptor superfamily[13]. They are mainly used as insulin-sensitizing drugs in patients with type 2 diabetes mellitus. Increasing evidence shows that TZDs not only improve insulin resistance but also have a broad spectrum of pleiotropic vascular effects [14]. Being activated by TZDs, PPAR-γ can heterodimerize with retinoic X receptor and recognize PPAR-response element in the promoters of target genes to regulate their expression [15]. The expression of PPAR-γ, initially thought to be restricted to adipose tissue, now has been documented in multiple vascular cell types, including endothelial cells[16], smooth muscle cells[17,18], and monocytes/macrophages[19], and regulates the gene expression of key proteins involved in vascular inflammation, cell proliferation and apoptosis.

Recently, considerable evidence points to a role of PPAR-γ and its agonists in inhibiting VSMC proliferation and preventing vascular remodeling in hypertension [20,21], restenosis [22,23], and atherosclerosis [23,24] in both early clinical trials and animal experiments. Much less is known about its underlying mechanisms. In this study, we aimed to elucidate whether rosiglitazone could inhibit Ang II-mediated proliferation in VSMCs by interfering with the Ang II/KLF5 signaling pathway.

## Materials and Methods

### Regents

Dulbecco’s modified Eagle’s medium (DMEM), fetal bovine serum (FBS), penicillin and streptomycin were from GIBCO BRL (Carlsbad, CA). 3-(4,5)-dimethylthiahiazo (-z-y1)-3,5-di- phenytetrazoliumromide, penicillin, streptomycin, Ang II, PD123319, 15-Deoxyprostaglandin J2 (15d-PGJ2), GW9662, bisphenol A diglycidyl ether (BADGE) PD98059 were from Sigma (St. Louis, MO, USA). Rosiglitazone, pioglitazone and losartan were from Alexis (Lausen, Switzerland). Polyclonal anti-rat KLF5, -cyclin D1, -phospho-protein kinase C (PKC)ε, -phospho-PKCζ, -PKCζ, -β-actin and-TBP antibodies were from Santa Cruz Biotechnology (Santa Cruz, CA, USA). Antibodies against extracellular signal-regulated kinase 1/2 (ERK1/2) and phospho-ERK1/2 were from Cell Signaling Technology (Beverly, MA, USA). Rabbit polyclonal antibody against PPAR-γ was from Upstate Inc. (Chicago, IL, USA). Small-interfering RNA (siRNA) specific for PPAR-γ, KLF5 and PKCζ (siGENOME, M-080081-00-0010, M-098194-01-0005 and M-090850-01-0005), negative control siRNA (NC siRNA) (siGENOME, D-001206-13-05) and DharmaFECT 2 transfection reagent (T-2002-02) were obtained from Dharmacon (Lafayette, CO, USA). Cell Cycle Phase Determination Kit was from Cayman Chemical (USA). NE-PER Nuclear and Cytoplasmic Extraction kit was from Pierce (IL, USA), TRIzol kit and SuperScript III Platinum SYBR-Green Two-Step qRT-PCR kit were provided by Invitrogen Corp. (Carlsbad, CA,USA). DNA-free kit was from Ambion (Austin TX, USA). Agarose gels were from Spanish Biochemicals (Pronadisa, Madrid, Spain). Reagents for the enhanced chemiluminescence were from Pierce Corp. (Rockford, IL, USA).

### *In vivo* experiments

The study was approved by the Institutional Animal Research and Ethics Committee of Xi’an Jiaotong University (SCXK2007-001) and was conducted in accordance with the National Institutes of Health (NIH) Guide for the Care and Use of Laboratory Animals (NIH Publication No. 85–23, revised 1996). Male Sprague-Dawley rats (200–220 g) were divided into 4 groups for treatment (n = 8): control, Ang II, Ang II+rosiglitazone and rosiglitazone alone. Rats were anesthetized with methoxyflurane, and then osmotic minipumps (model 2004, Durect Corp., Cupertino, CA) were inserted subcutaneously to deliver Ang II (150 ng/kg/min) or the same volume of 0.9% saline for controls for 28 days. Rosiglitazone was administrated (5 mg/kg/d in drinking water) for 29 days, starting the day before Ang II infusion. Systolic blood pressure was measured by the tail-cuff method. The animals were killed by injecting an excess amount of pentobarbital. One portion of the aorta was dissected and cleaned of fat, then frozen in liquid nitrogen for RNA extraction. Another portion was fixed in 4% formaldehyde solution and embedded in paraffin for immunohistochemical analysis.

### Vascular morphology

Cross-sections of thoracic aorta segments collected at the time of sacrifice were paraffin-embedded and stained with alizarin blue. Morphometric assessment was performed using the Qwin 550 quantitative image analysis system (Leica, German). Equally spaced (every 45°) measurements of lumen diameter (4 measurements) and wall thickness (8 measurements) were made. The averaged wall thickness was divided by the averaged lumen diameter to calculate a final wall/lumen ratio.

### Immunohistochemistry

Primary antibodies against KLF5 (1:400) and cyclin D1 (1:250) were added to paraffin-embedded sections of rat thoracic aortas and incubated overnight at 4°C. Biotinylated and affinity-purified IgG (Zymed, USA) was used as a secondary antibody and incubated for 1 hr at 37°C. A streptavidin-enzyme conjugate was sequentially added for 20 min and incubated with substrate 3’, 3’ -diaminobenzidine (DAB), followed by haematoxylin nuclear counterstaining. Negative controls were without the primary antibody. Quantification results detected by Leica QWin550CW Image Acquireing & Analysis System (Leica, German) are presented as gray scale levels.

### Cell culture

VSMCs were obtained from thoracic aortas of Sprague-Dawley rats as previously described [25] and cultured in DMEM containing 10% FBS, penicillin (100 U/ml) and streptomycin (100 U/ml). VSMCs between passages 3 to 7, confirmed positive (99%) for smooth muscle α-actin immunostaining, were used in the experiments. For subsequent experiments, cells at 80% confluence in culture wells were growth-arrested by serum starvation for 48 hrs.

### Cell proliferation assay

Cell proliferation was determined using the cell counting kit-8 (CCK-8, Dojindo, JAPAN) according to the manufacturer's protocol. Briefly, 2500 cells/well were plated in a 96-well plate. After treatment, 10 μl of CCK-8 solution was added to each well and incubated for 3 hrs. The cell viability in each well was determined by reading the optical density at 450nm.

### Small-interfering RNA

**Fig 1:**
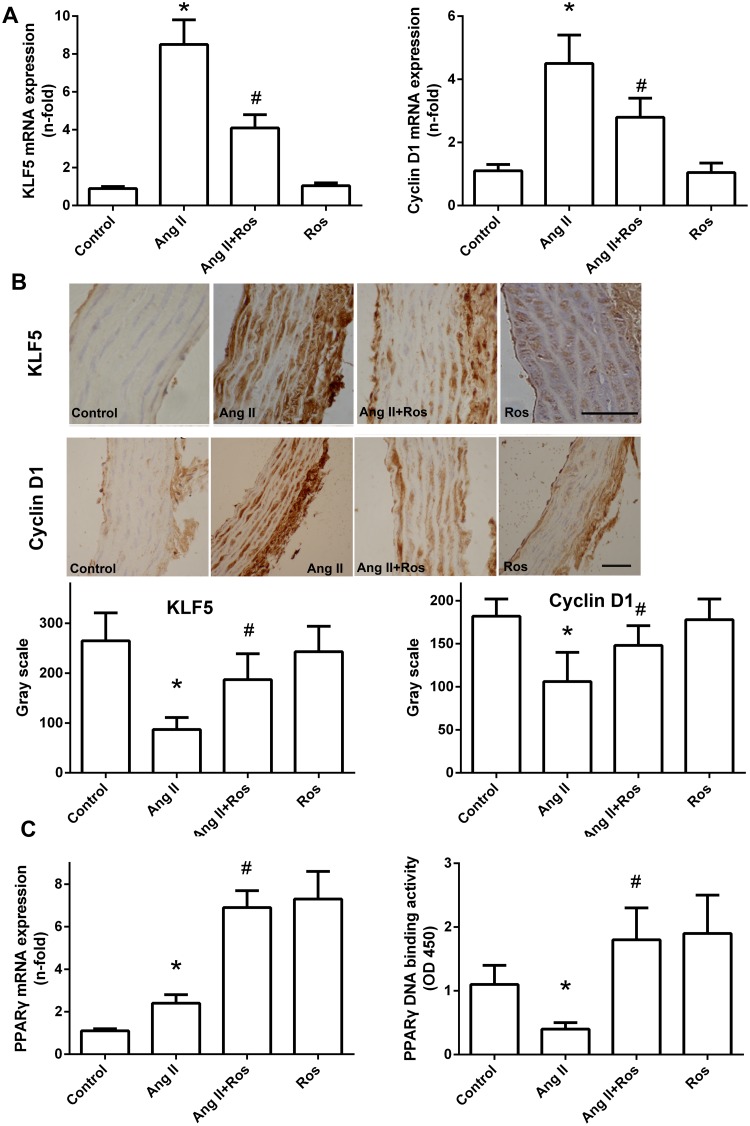
*In vivo* induction of KLF5 in response to Ang II and altered expression in response to rosiglitazone. (A) KLF5 and cyclin D1 expression as measured by real-time RT-PCR in the thoracic aorta of Ang II-infused rats (150 ng/kg/min) with or without rosiglitazone. Values are expressed as fold induction compared with control group. (B) Immunohistochemical analysis of KLF5 and cyclin D1 expression in each group. Images are representative of 6 animals studied in each group. Scale bar = 50 μm. Quantification results are presented as gray scale levels and mean ± S.E.M. data of 24 measurements in 6 slides. (C) PPAR-γ mRNA expression was analyzed by real-time RT-PCR. (D) PPAR-γ activation was analyzed by DNA-binding assay. *P<0.*05* vs. control; #*P<0*.*05* vs. Ang II alone. Ros: rosiglitazone.

The VSMCs (5×10^6^) were seeded into six-well plates and were grown until 60–80% confluent. The cells were transiently transfected with 150 pM of PPAR-γ siRNA, KLF5 siRNA, PKCζ siRNA or NC siRNA and using DharmaFECT 2 transfection reagent according to the manufacturer’s instructions. After 48 hrs, PPAR-γ mRNA, KLF5 mRNA and PKCζ mRNA levels were detected by quantitative real time-PCR respectively. Knock down efficiency of PPAR-γ, KLF5 and PKCζ were showed in S1 Fig 1 as determined by quantitative real-time PCR and western blot. Transfection rates of 60–70% of the cells were accepted for all the experiments.

### Real-time RT-PCR

Total RNA was extracted by use of TRIzol reagent, and DNA was removed by use of the DNA-free kit (Ambion, Austin TX, USA). Real-time qRT-PCR with SYBR involved use of the SuperScript III Platinum Two-Step qRT-PCR Kit on an ABI PRISM 7000 sequence detection PCR system (Applied Biosystems, Foster City, CA, USA) according to the manufacturers’ instructions. Primers were designed with use of Beacon Designer v4.0 (Premier Biosoft, USA; Table 1). Results are expressed as fold difference in gene expression to that of GAPDH by the 2^-ΔΔCT^ method[26]. To validate our real-time PCR protocol, gene-specific standard curves for each gene and GAPDH were generated from serial 10-time dilutions of the cDNA. Expression slopes of each gene were similar to that of GAPDH. A melting-curve analysis was also performed to check for the absence of primer dimers.

**Table 1:** Primer Sequence Used for qRT-PCR.

Gene	Primer sequence	Accession No
PPAR-γ	5’-TGGAG-CCTAA-GTTTG-AGTTT G-3’	**NM_013124**
	5’-ATCTT-CTGGA-GCACC-TTGG-3’	
KLF5	5’-AGCCA-C CAGAG-CGAAT-CC-3’	**NM 053394**
	5’-GCCAG-CCATA-GAGACATTAA-GG-3’	
Cyclin D1	5’- ACCAA-TCTCC-TCAAC-GACC -3’	**NM_171992**
	5’- TTGTT-CTCAT-CCGCC-TCT -3’	
AT1R	5’-CTCAG-CCACC-TAACT-TCC-3’	**NM_030985**
	5’-TTGTG-TTCCA-GAGTA-GCC-3’	
GAPDH	5'-GCCTT-CTCCA-TGGTG-GTGAA-3'	**NM_017008**
	5'-GGTCG-GTGTG-AACGG-ATTTG-3')	

### Western blot analysis

Protein samples (20 μg) were resolved on 10% SDS-PAGE and transferred to a polyvinylidene difluoride membrane in a semi-dry system (Bio-Rad, Hercules, CA). The membranes were incubated with specific antibodies against KLF5 (1:500), ERK1/2 (1:500), p-ERK1/2 (1:500), p-PKCε (1:400), p-PKCζ (1:400), PKCζ(1:400), TBP (1:1000) and β-actin (1:2000). Signals were revealed with chemiluminescence and visualized by exposure to x-ray films. Optical densities of bands were scanned and quantified with the Gel Doc 2000 system (Bio-Rad). Results are expressed as fold increase as compared with that of the control.

### Immunofluorescene

Cells were fixed with 4% paraformaldehyde, permeablized with 0.3% Triton-X 100, and incubated with primary antibody at 4°C overnight, and then subjected to Cy3 labeled secondary antibody for 1 hr at room temperature. Visualization was performed by using Zeiss LSM 510 laser scanning confocal microscope.

### DNA-binding assay

Nuclear proteins were extracted by use of the NE-PER kit. DNA-binding activity of PPAR-γ and early growth response (Egr) transcription factor was detected by an ELISA-based method with PPAR-γ (Cayman Chemical, USA) and Egr (Genlantis, USA) transcription assay kits according to the manufacturers’ instructions. Briefly, 10 μg nuclear protein was added to the 96-well plate pre-coated with PPAR or Egr specific double-strand DNA containing the sequence for peroxisome proliferators-response element (PPRE) or Egr response element, and then incubated over night at 4°C. Bound PPAR-γ or Egr was detected by the specific PPAR-γ or Egr antibody. A horseradish peroxidase-conjugated secondary antibody was then added for colorimetric reading.

### Statistical analysis

Results are expressed as mean ± s.e.m. Statistical significance between groups was assessed by one-way ANOVA, followed by post-hoc Duncan multiple comparisons, with use of SPSS v11.5 (SPSS Inc., Chicago, IL). A *P* <0.05 was considered statistically significant.

## Results

### Systolic blood pressure, body weight and vascular morphology changes in Ang II-infused rats

After the 28-day treatment, body weights were similar in all groups (Table 2). Ang II infusion induced a substantial increase in systolic blood pressure, (*p<*0.05 versus Control), which was attenuated by rosiglitazone (*p<*0.05 versus Ang II and control). The Lumen diameter (L), Media thickness (M) showed no difference in each group (*P>*0.05). (S2 Fig) Media/lumen (M/L) ratio was higher in Ang II group when compared with control (*P<*0.05), whereas rosiglitazone significantly decreased M/L ratio when compared with Ang II group (*P<*0.05).

**Table 2:** SBP, BW and vascular morphology changes in Ang II-infused rats.

Parameter	Control	Ang II	Ang II +Ros	Ros
BW(g)	323±4	325±6	326±7	325±4
SBP(mmHg)	110±4	165±9*	127±5#	114±3
Lumen diameter(mm)	1.68±0.19	1.71±0.17	1.69±0.21	1.67±0.14
Media thickness (μm)	126±9	131±12	128±11	127±11
Media/lumen ratio	0.078±0.011	0.085±0.014*	0.075±0.016^#^	0.079±0.009

*P<0.*05* vs. control

^#^ *P<0*.*05* vs. Ang II.

### Rosiglitazone attenuated KLF5 and cyclin D1 expression in Ang II-infused rats

In rats infused with Ang II for 28 days, KLF5 mRNA expression was induced in the aortas, and this change was significantly inhibited by rosiglitazone treatment (Fig 1A). To define the cellular localization and protein expression of KLF5 after the appropriate treatment, we examined the expression of KLF5 protein by immunohistochemistry within the aortas (Fig 1B). Robust KLF5 expression was observed in the aortic VSMCs of Ang II-treated animals, which was significantly decreased by rosiglitazone treatment. Rosiglitazone alone had no effect on KLF5 expression.

We then evaluated whether rosiglitazone diminished VSMC proliferation in parallel with KLF5 downregulation by measuring cyclin D1 expression in the aorta. Infusion of Ang II increased the expression of cyclin D1 within the thoracic aorta of Ang II-infused rats (Fig 1A and 1B). Rosiglitazone significantly inhibited this Ang II-mediated effect. Expression and activation of PPAR-γ in the thoracic aorta were identified with real-time RT-PCR and DNA binding assay. As show in (Fig 1C). The increase of PPAR-γ mRNA expression and activation was observed after rosiglitazone treatment.

### PPAR-γ agnosits inhibited Ang II induced cell proliferation in VSMCs

**Fig 2:**
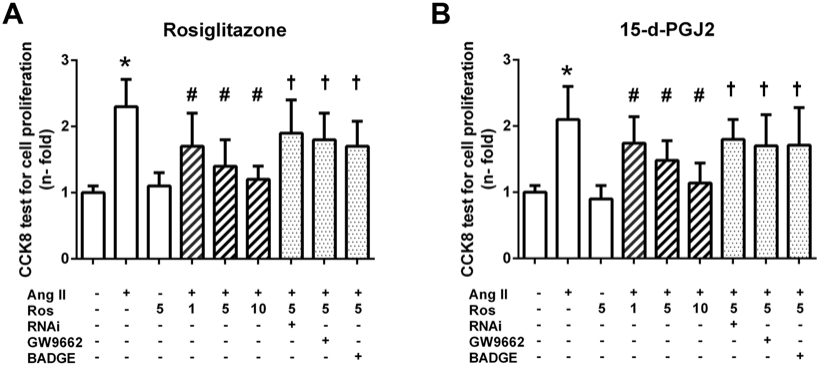
Effects of PPAR-γ activation on cell proliferation and cell cycle in Ang II-stimulated vascular smooth muscle cells (VSMCs). Growth-arrested VSMCs were switched to fresh medium containing 0.5% of serum when pretreated with or without PPAR-γ antagonist GW9662 (3 μM), BADGE (1 μM) for 30 min or PPAR-γ specific siRNA for 48 hrs prior to the addition of rosiglitazone (1, 5 and 10 μM) or PPAR-γ nature ligand 15-d-PGJ2 (1, 5 and 10 μM). Ang II (0.1 μM) was then added for 24 hrs. (A). CCK8 assays (values represent the mean ± S.E.M. (n = 12) were carried out to assess cell proliferation. Ros = rosiglitazone; 15d = 15-d-PGJ; Ang II = Angiotensin. *P<0.*05* vs. control; #*P<0*.*05* vs. Ang II; †*P<0*.*05* vs. Ang II + Ros.

As Seen in Fig 2, Ang II (0.1 μM) induced a significant increase in the cell proliferation (*P<*0.001 versus control). Pretreatment of the cells with PPAR-γ agonists rosiglitazone (Fig 2A) and 15-d-PGJ2 (Fig 2B) markedly inhibited this effect in a dose-dependent manner (*P<*0.05 versus Ang II). Moreover, this effect was substantially attenuated by the PPAR-γ antagonists GW9662 (3 μM) or BADGE (1 μM), and PPAR-γ specific siRNA (*P*<0.05 versus Ang II + Ros).

### PPAR-γ agonists inhibited Ang II-induced KLF5 and cyclin D1 expression in VSMCs

**Fig 3:**
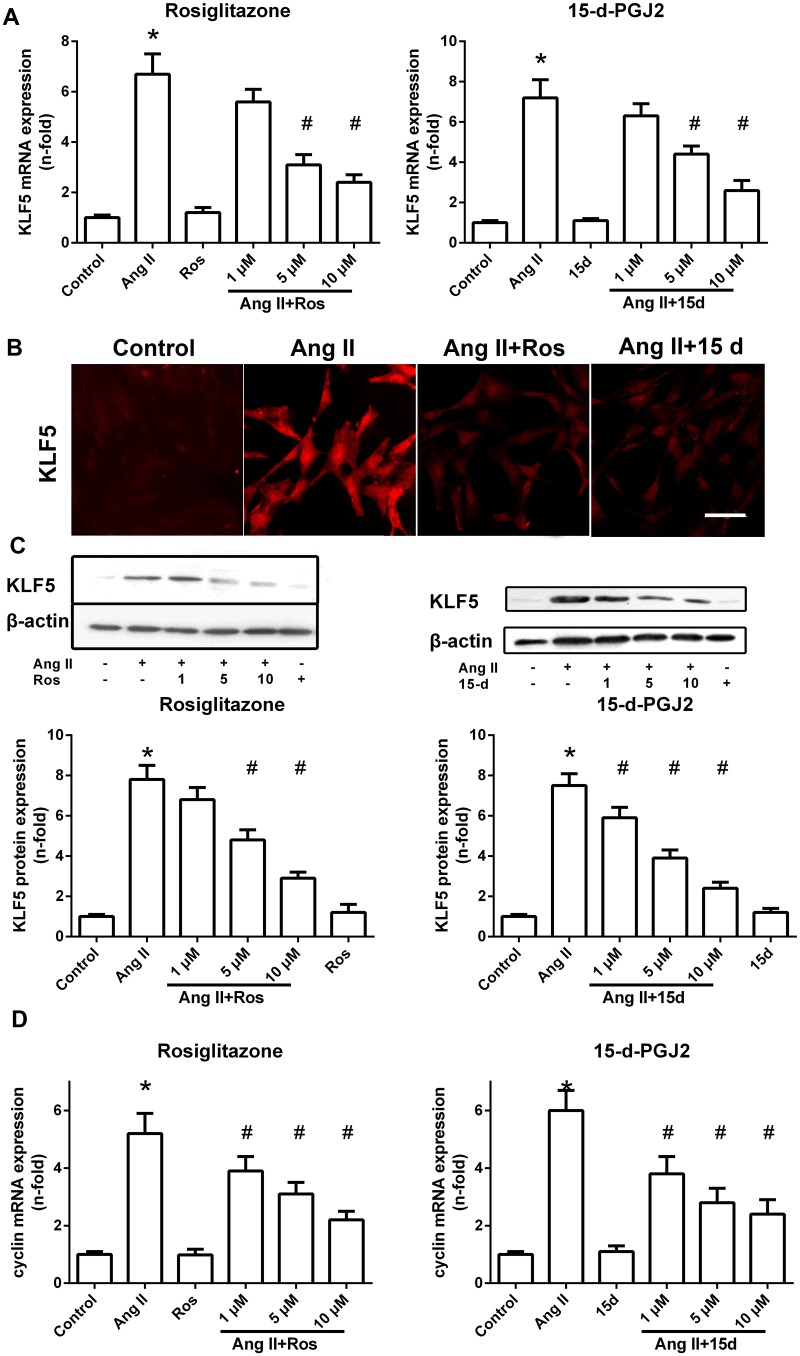
PPAR-γ agonists inhibit KLF5 and cyclin D1 expression in Ang II-stimulated VSMCs. Cells were pretreated with increasing concentrations of rosiglitazone (1, 5 and 10 μM) or 15-d-PGJ2 (1, 5 and 10 μM) for 1 hr and then stimulated with Ang II (0.1 μM) for 24 hr. (A) Real-time RT-PCR analysis of KLF5 mRNA expression in VSMCs. Results are expressed as fold increase over control group, and data are mean ± S.E.M. of 3 independent experiments. GAPDH served as an internal control. (B) Immunocytochemical staining of KLF5 staining in VSMCs. Scale bar: 100 mm. (C) Western blot analysis of KLF5 protein expression in VSMCs. Results are representative of 3 independent experiments. β-actin served as an internal control. Data are mean ± S.E.M. of 3 experiments. (D) Real-time RT-PCR analysis of cyclin D1 mRNA expression in VSMCs. Results are expressed as fold increase over control group, and data are mean ± S.E.M. of 3 independent experiments. GAPDH served as an internal control. (*P <0.*05* vs. control; #*P <0*.*05* vs. Ang II). Ros = rosiglitazone; 15d = 15-d-PGJ; Ang II = Angiotensin.

Results from real-time reverse transcription-PCR analysis showed that both rosiglitazone and 15d-PGJ2 substantially reduced the increased levels of KLF5 mRNA in response to Ang II in a dose-dependent manner (Fig 3A) (P<0.05 versus Ang II). Notably, rosiglitazone and 15-d-PGJ2 had no significant effect on the basal expression of KLF5 in VSMCs (P>0.05 versus control). Similar results were found by using immunofluorescene (Fig 3B) and western blot analysis for detecting of total (Fig 3C) and nuclear (S3 Fig) KLF5 protein in VSMCs. In addition, as assessed by real-time RT-PCR, rosiglitazone and 15d-PGJ2 also dose dependently inhibited Ang II-induced cyclin D1 mRNA expression, which was well known as a target gene of KLF5 (Fig 3D).

### Relationship between the effect of rosiglitazone on Ang II-induced cell proliferation and KLF5 in VSMCs

**Fig 4:**
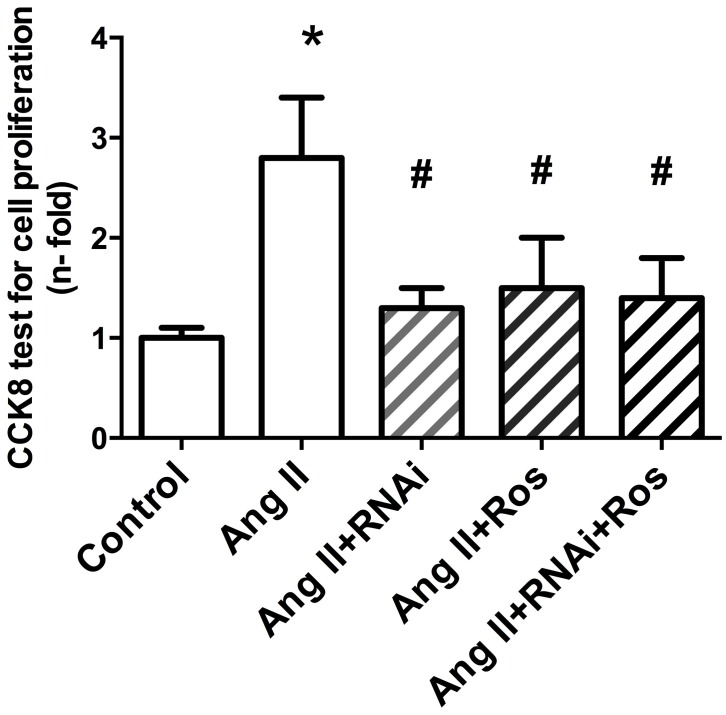
Relationship between effect of rosiglitazone on Ang II-induced cell proliferation and KLF5 expression in VSMCs. VSMCs were pretreated with KLF5 siRNA for 1 hr prior to the addition of rosiglitazone (5 μM) for 1 hr, and subsequently stimulated with Ang II (0.1 μM) for 24 hrs. CCK8 assays (values represent the mean ± S.E.M. n = 12) were carried out to assess cell proliferation. *P<0.*05* vs. control; #*P<0*.*05* vs. Ang II; †*P<0*.*05* vs. Ang II + Ros.

As mentioned above, rosiglitazone is able to inhibit Ang II-induced cell proliferation and KLF5 expression in VSMCs in vivo and in vitro. To confirm whether rosiglitazone suppresses Ang II-induced proliferation responses via KLF5, VSMCs were pretreated with KLF5 siRNA for 48 hrs prior to the addition of rosiglitazone (5 μM) for 1 hr, and subsequently stimulated with Ang II (0.1 μM) for 24 hr. As shown in Fig 4, When compared with the control, stimulating the cells with Ang II induced cell proliferation (P<0.05 versus control), whereas the KLF5 siRNA and rosiglitazone partially reversed this effect in VSMCs (P<0.05 versus Ang II). Moreover, rosiglitazone did not further reverse the effect of Ang II induced cell proliferation when pretreatment of the cells with the KLF5 siRNA (P>0.05 versus Ang II+RNAi). These results indicated that the modulatory effect of rosiglitazone on cell proliferation in Ang II-stimulated VSMCs is at least in part related to KLF5.

### Suppression of KLF5 expression is mediated by PPAR-γ activation

**Fig 5:**
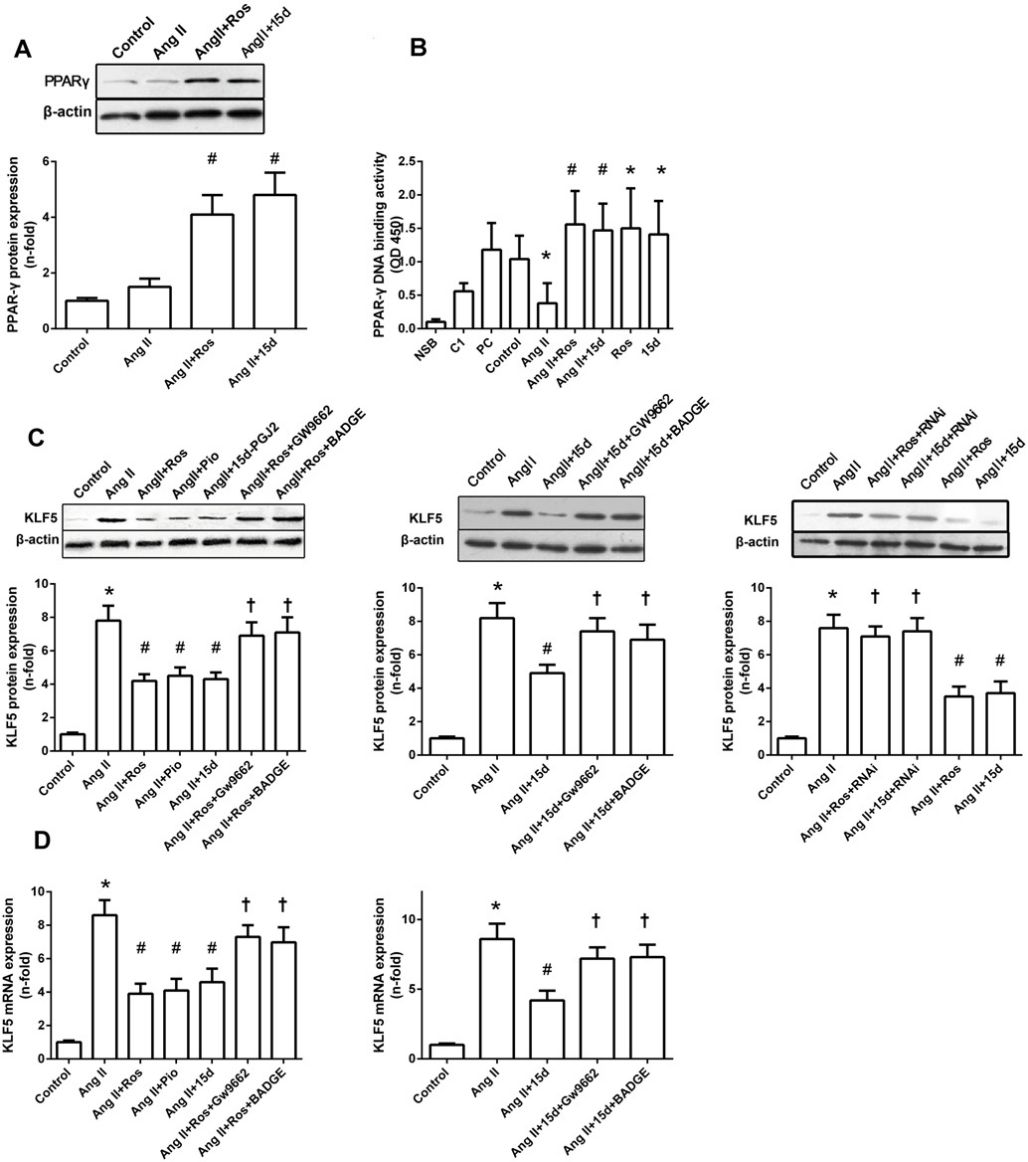
Suppression of KLF5 expression is mediated by PPAR-γ activation. Cells were pretreated with or without PPAR-γ antagonist GW9662 (3 mM) or BADGE (1 μM) for 30 min or PPAR-γ specific siRNA for 48 hrs prior before the addition of PPAR-γ activator rosiglitazone (Ros) (5μM), 15-d-PGJ2 (15D) (5μM) or pioglitazone (Pio) (50 μM) for 1 hr, and subsequently stimulated with Ang II (0.1 μM) for 24 hrs. (A) Representative immunoblots for PPAR-γ and β-actin from 3 separate experiments. PPAR-γ protein expression is shown as fold increase over control group. Results are mean ± S.E.M. β-actin was used as an internal control. (B) PPAR-γ activation was analyzed by DNA-binding assay. NSB: non-specific binding, C1: competitor binding, PC: positive control binding. Results are mean ± S.E.M. of 3 independent experiments, expressed as OD 450. (C) Western blot analysis of KLF5 protein expression in VSMCs. Representative western blot (upper panel), and data are mean ± S.E.M. (bottom panel) of 3 independent experiments. Results are expressed as fold increase over control group. β-actin served as an internal control. (D) Real-time RT-PCR analysis of KLF5 mRNA expression in response to different treatment in VSMCs. Results are fold increase over control, and data are mean ± S.E.M. of 3 independent experiments. GAPDH served as an internal control. (*P<0.*05* vs. control; # *P<0*.*05* vs. Ang II; †*P<0*.*05* vs. Ang II + Ros).

As shown in Fig 5A, Ang II treatment for 24 hrs elicited a trend to an induction of PPARγ mRNA expression, although it did not achieve statistical significance (P>0.05 versus control). Moreover, Ang II significantly decreased PPAR-γ DNA binding activity as compared with the control (*P<*0.05; Fig 5B). Exposure to rosiglitazone and 15d-PGJ2 for 24 hrs resulted in a significant increase in the DNA-binding activity to PPRE. Moreover, pretreatment with rosiglitazone or 15d-PGJ2 substantially increased PPAR-γ DNA-binding activity in comparison with Ang II treatment (*P<*0.05 versus Ang II).

As shown in Fig 5C and 5D, western blot and real time RT-PCR analysis showed that rosiglitazone, 15d-PGJ2 and pioglitazone were all able to suppress Ang II induced KLF5 mRNA and protein expression. In contrast, Pretreated cells with PPAR-γ antagonists (GW9662 and BADGE) or PPAR-γ specific siRNA diminished these effects (Fig 5C and 5D). These results clearly indicated that the effect of PPAR-γ agonists on Ang II-induced KLF5 expression is mediated at least in part by PPAR-γ activation.

### Effect of rosiglitazone on ANG II receptors and signaling in regulating KLF5 expression

**Fig 6:**
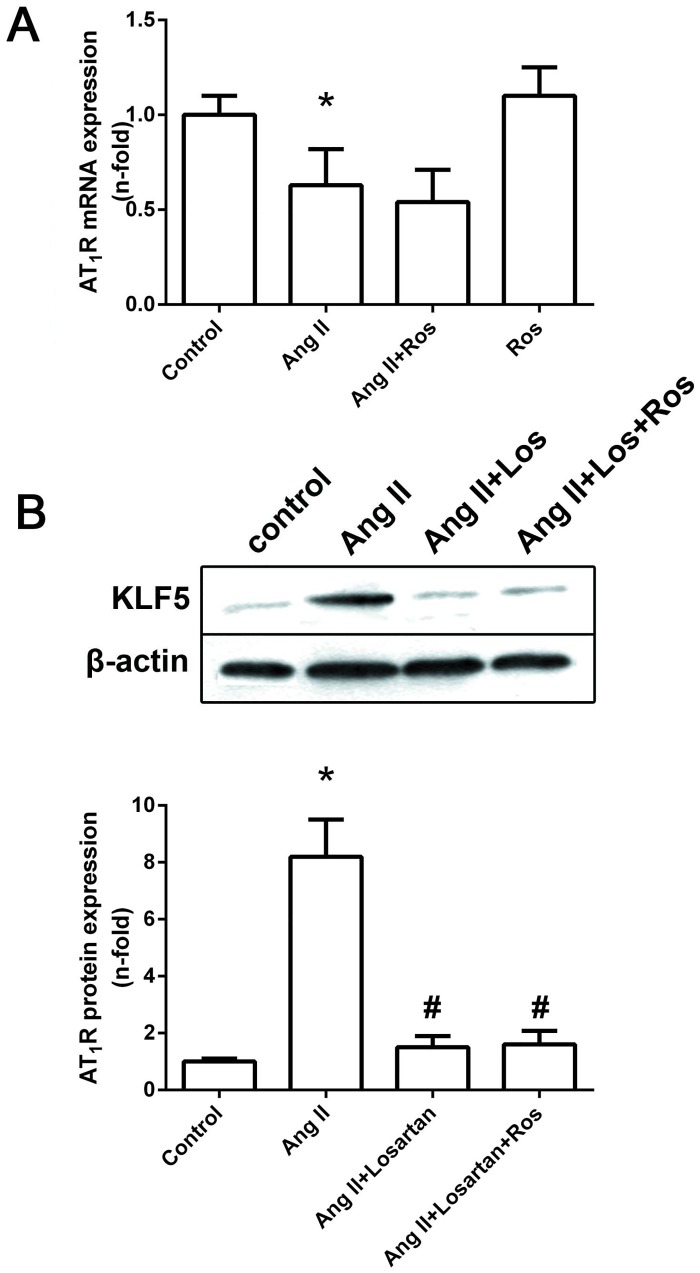
Blockade of AT_1_ with losartan was not involved in the inhibitory effect of rosiglitzone on Ang II-induced KLF5 expression in VSMCs. Western blot analysis (upper panel), and data are mean ± S.E.M. (bottom panel) of 3 independent experiments. Results are expressed as fold increase over control group. β-actin served as an internal control (*P<0.*05* vs. control; #*P<0*.*05* vs. Ang II).

To clarify whether blockade of the Ang II receptor signaling was involved in the inhibitory effect of rosiglitazone on Ang II-induced KLF5 expression in VSMCs, VSMCs were subjected to losartan (1 μM), PD123319 (10 μM) for 30min, followed by treatment of rosiglitazone (5 μM) for further 1 hr, and finally stimulated with Ang II (0.1 μM) for 24 hrs. As seen in Fig 6, AT_1_ antagonist losartan inhibited Ang II-induced KLF5 expression in VSMCs, whereas the AT2 antagonist PD123319 had no effect (data not shown). Although rosiglitazone alone also inhibited Ang II-induced KLF5 expression in VSMCs, it did not have any additional effects to AT_1_ blockades, suggesting that the inhibitory effects of PPAR-γ agonist on Ang II stimulatory events might be downstream of the AT_1_ receptor.

**Fig 7:**
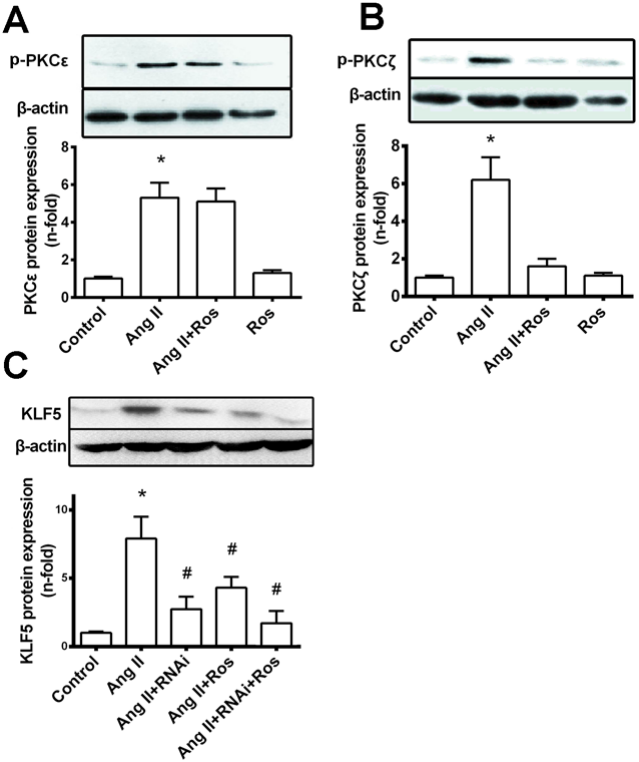
PKC signaling pathway in the effect of PPAR-γ agonist on Ang II-induced KLF5 expression. (A) and (B). VSMCs were pretreated with rosiglitazone (5 μM) for 1hr, and incubated with Ang II (0.1 μM) for 30 min; phosphorylated PKCε and PKCζ were then detected by western blot. Representative western blot of p-PKCε and p-PKCζ, and data of mean ± S.E.M. of 3 experiments (bottom panel) were shown. (C). VSMCs were pretreated with PKCζ siRNA for 48 hrs prior to the addition of rosiglitazone (5 μM) for 1 h, and subsequently stimulated with Ang II (0.1 μM) for 24 h. A representative western blot of KLF5, and data of mean ± S.E.M. of 3 experiments (bottom panel) were shown. All results are expressed as fold increase over control group. β-actin served as an internal control. (*P<0.*05* vs. control; #*P<0*.*05* vs. Ang II).

We then explored the regulation of AT_1_R/PKC signaling in the inhibitory effect of rosiglitazone on Ang II-induced KLF5 expression in VSMCs. As shown in Fig 7A and 7B, phosphorylation of both PKCε and PKCζ was induced by Ang II (0.1 μM) treatment for 30 min, whereas pretreatment of cells with rosiglitazone (5 μM) abrogated only PKCζ phosphorylation (by more than 80%). Moreover, as seen in Fig 7, PKCζ specific siRNA significantly diminished Ang II induced KLF5 expression (P<0.05 vs. Ang II) and rosiglitazone treatment potentiated the inhibitory effect of the PKCζ RNAi on KLF5 expression. Taken together, these results indicate that the inhibitory effect of PPAR-γ agonist on Ang II-induced KLF5 expression in VSMCs is at least part from Ang II/ PKCζ pathway.

**Fig 8:**
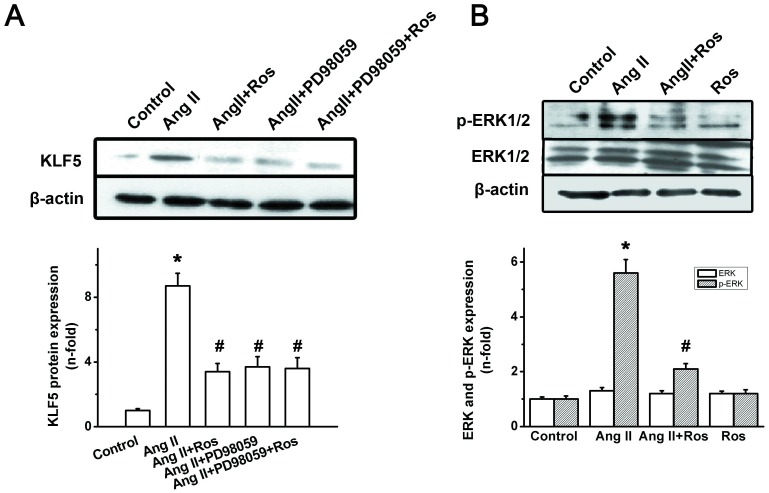
ERK signaling pathway in the effects of PPAR-γ agonist on Ang II-mediated KLF5 expression. (A) VSMCs were subjected to ERK1/2 inhibitor PD098059 (1 μM) for 30min, followed by treatment of rosiglitazone (5 μM) for further 1 h, and finally stimulated with Ang II (0.1 mM) for 24 h. A representative western blot of KLF5 (upper panel), and data of mean ± S.E.M. of 3 experiments (bottom panel) were shown. (B) VSMCs were pretreated with rosiglitazone (5 μM) for 1h, and incubated with Ang II (0.1 μM) for 30 min, phosphorylated ERK1/2 were then detected by western blot. A representative western blot of ERK1/2 and p-ERK1/2, and data of mean ± S.E.M. of 3 experiments (bottom panel) were shown. Results are expressed as fold increase over control group. β-actin served as an internal control. (*P<0.*05* vs. control; #*P<0*.*05* vs. Ang II.)

Finally, we observed whether the blockade of ERK1/2 was required for the inhibitory effect of rosiglitazone on Ang II-induced KLF5 expression (Fig 8). VSMCs were pretreated with the specific ERK1/2 inhibitor PD98059 (1 μM) for 30min before the addition of rosiglitazone (5 μM) for 1 hr, and then stimulated with Ang II (0.1 μM), As illustrated in Fig 8A, Erk1/2 inhibitor PD98059 significantly attenuated Ang II induced KLF5 expression, whereas, rosiglitazone did not have any additional effects on ERK1/2 blockade, which suggests that Ang II/ERK1/2 signaling may be involved in the inhibitory effect of PPAR-γ agonist on Ang II-induced KLF5 expression. Furthermore, as shown in Fig 8B, ERK1/2 phosphorylation induced by Ang II (0.1 μM) treatment for 30 min was significant impaired by rosiglitazone. These results suggest that Ang II/ERK1/2 signaling might also contribute to the inhibitory effect of rosiglitazone on Ang II-induced KLF5 expression in VSMCs.

### Inhibitory effect of rosiglitazone on EGR transcription activity

**Fig 9:**
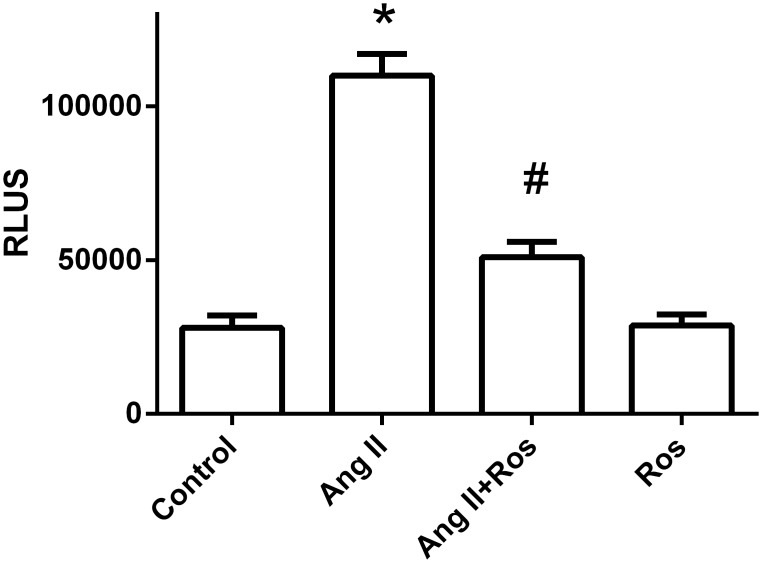
Effect of PPAR-γ agonist on Egr activity. Egr activation was analyzed by DNA-binding assay. Results are mean ± S.E.M. of 3 independent experiments, expressed as relative light units (RLUs). (*P<0.*05* vs. control; #*P<0*.*05* vs. Ang II).

To evaluate whether KLF5 expression inhibition by rosiglitazone is mediated by the downregulation of Egr activation, we tested the effect of rosilgitazone on Egr DNA-binding activity. As shown in Fig 9, rosiglitazone significantly abolished Ang II-induced Egr DNA-binding activity (P<0.05 versus Ang II).

## Discussion

**Fig 10:**
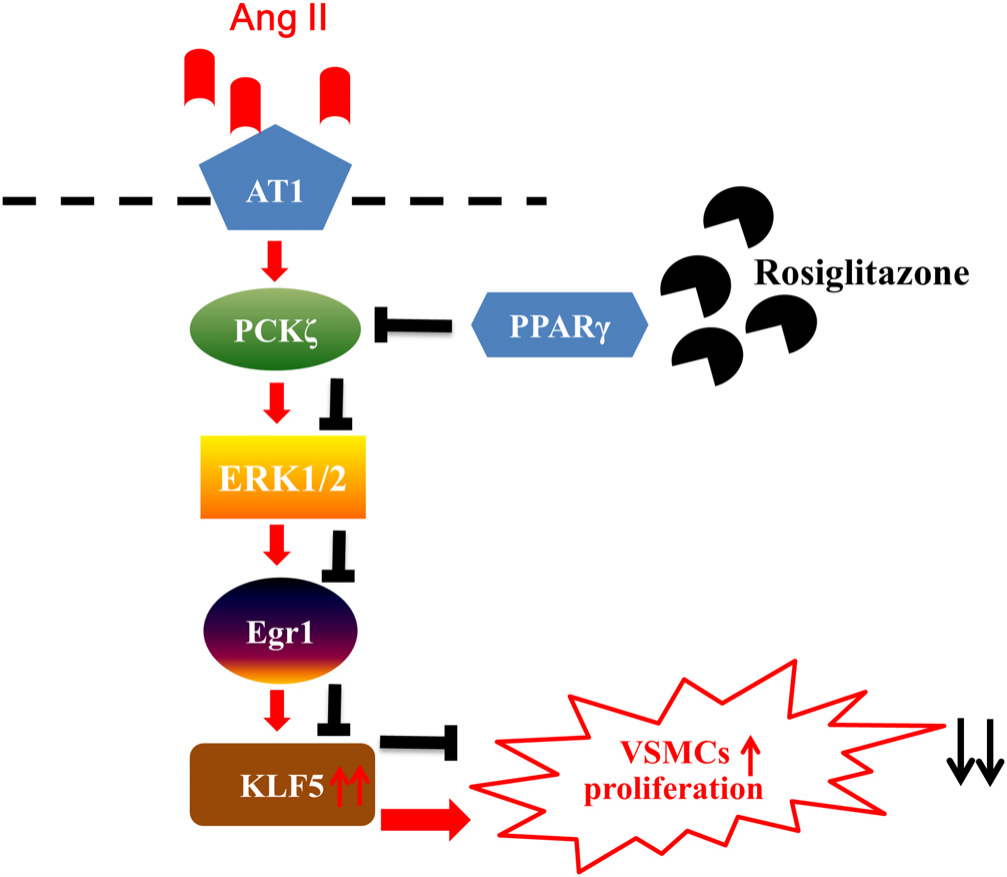
Agonist-induced activation of PPAR-γ suppresses Ang II-induced KLF5 expression, likely by interfering with the Ang II/PKCζ/ERK1/2/Egr pathway.

PPAR-γ activation by its agonists such as TZDs, has been demonstrated previously to inhibit VSMC proliferation and prevent vascular remodeling in hypertension, restenosis, and atherosclerosis in both early clinical trials and animal experiments[21–24,27]. Understanding the molecular mechanisms responsible for their beneficial efficacy in cardiovascular disease provides an important basis for the future development of PPAR-γ agonist in the treatment of vascular diseases. A considerable body of evidence indicates the pivotal role of KLF5 in cell proliferation, and inhibition of KLF5 expression has been demonstrated recently to inhibit neointima formation, atherosclerosis and hypertension related vascular remodeling [11,28]. In the present study, we investigated the regulation of KLF5 expression by the PPAR-γ agonist such as rosiglitazone, demonstrated that rosiglitazone-induced activation of PPAR-γ suppresses Ang II-induced KLF5 expression, likely by interfering with the Ang II/PKCζ/ERK1/2 pathway, and subsequently downregulated Egr transcriptional function (Fig 10). These findings demonstrate a previously unrecognized mechanism by which PPAR-γ agonists inhibit VSMC proliferation and document a novel evidence for the beneficial vascular effect of PPAR-γ activation.

In VSMCs, as the major target for Ang II in vascular remodeling, KLF5 is critically involved in the pathogenesis of cell proliferation and migration[11] and therefore may have a fundamentally significant contribution to the pathophysiological relationship between vascular remodeling and disorders [7]. Furthermore, our recent reports[10] and those of other researchers have shown that KLF5 in VSMCs is also induced by Ang II and plays a crucial role in Ang II-induced VSMC proliferation [9,12,29]. Consistent with the previous report, we observed a significant increase of cell proliferation and KLF5 induction by Ang II in VSMCs both *in vivo* and *in vitro*. Importantly, the present study provides the first evidence that the PPAR-γ agonist suppresses Ang II-induced KLF5 expression in VSMCs. Moreover, rosiglitazone did not further inhibit Ang II-induced cell proliferation in VSMCs when pretreatment of cells with KLF5 siRNA, thus suggesting that activation of PPAR-γ may intervene in the impact of Ang II on the KLF5 to inhibit VSMC proliferation.

Although TZDs have been reported to exert their effects through PPAR-γ-dependent and-independent mechanisms, the observed inhibition of Ang II-induced KLF5 expression we found is likely mediated by the activation of PPAR-γ, as demonstrated by several lines of evidence. Firstly, we confirmed previous data that Ang II decreased DNA-binding activity of PPAR-γ to PPRE, whereas pretreating cells with rosiglitazone and 15d-PGJ2 significantly upregulated PPAR-γ expression and the DNA-binding activity. Secondly, both a different synthetic TZD PPAR-γ agonist and a natural agonist, 15d-PGJ2, mimicked the effect of the PPAR-γ activation on KLF5 expression with equivalent inhibitory potency. Finally, we tested both the specific pharmacological inhibitors of PPAR-γ (BADGE and GW966) and the PPAR-γ specific siRNA on KLF5 suppression by PPAR-γ agonist. The findings that PPAR-γ inhibitions by BADGE, GW9662 and RNAi overcame KLF5 suppression by PPAR-γ agonist demonstrate an essential involvement of PPAR-γ in this effect.

Ang II acts through its binding to a specific AT_1_ receptor that regulates KLF5 expression. Previous studies demonstrated that PPAR-γ activation might transcriptionally regulate AT_1_ expression [30]. We showed that rosiglitazone cotreatment had little effect on AT_1_ receptor mRNA expression in Ang II-challenged VSMCs, which indicates that this mechanism may not be involved in the inhibitory effect of PPAR-γ agonists on Ang II-induced KLF5 expression. Our finding is in accordance with those of Takeda[31] and Sugawara[32], who demonstrated that 24-hr treatment with rosiglitazone had the little effect on AT_1_ expression in VSMCs as compared with untreated cells.

Several studies have demonstrated distinct roles of PKC isoforms in regulating cell proliferation. PKCα and –δ activation may inhibit cell proliferation [33,34]; however, PKCζ and –ε were reported to induce VSMC proliferation in response to many simulators, including Ang II [35,36]. In light of previous research [9,29], Ang II-induced KLF5 expression in VSMCs is PKC dependent, so we explored the possibility whether PPAR-γ could inhibit Ang II-induced KLF5 induction by suppressing PKC activity. As indicated by their phosphorylation, both PKCζ and –ε were indeed activated by Ang II, but rosilgitazone inhibited only the PKCζ activation. Moreover, siRNA PKCζ significantly downregulated Ang II induced KLF5 expression in VSMC. Therefore, the decrease in Ang II-induced KLF5 expression and cell proliferation by PPAR-γ agonists we observed is, at least in part, by interfering with the switch of PKCζ activation in the Ang II signaling pathway, and other PKC-independent pathway may also be involved. The interaction between the PPAR-γ and PKC pathway was also demonstrated in several other studies. PPAR-γ activated by TZDs inhibited glucose-induced activation of diacylglycerol—PKC signaling pathway in endothelial cells[37]. In PDGF-stimulated VSMCs, rosiglitazone and troglitazone significantly inhibited PKCδ activation [38]. Indeed a recent study suggested that rosiglitazone may negatively modulate high-glucose-induced signaling through PKCζ in mesangial cells [39].

ERK1/2 is downstream of the PKCζ signal pathway in regulating VSMC proliferation in response to Ang II[36] and is implicated in vascular diseases[40]. Our study demonstrated that PPAR-γ agonist markedly diminished Ang II-induced ERK1/2 phosphorylation. Blockade of ERK1/2 by PD98059 significantly diminished Ang II induced KLF5 expression. Moreover, rosiglitazone did not have any additional effects on KLF5 expression when ERK1/2 was blocked, which suggests that ERK1/2 signaling, at least in part, may be involved in the inhibitory effect of PPAR-γ agonist on Ang II-induced KLF5 expression in VSMCs. In accordance with our study, previous studies have demonstrated that PPAR-γ activation may inhibit Ang II induced ERK1/2 activation in endothelial cells[41,42] and VSMCs [43,44]. Recently, Kim et al. has showed the similar result that rosiglitazone might attenuate VSMCs proliferation at least by inhibit ERK1/2 activation and other cell signaling such as, mTOR-p70S6K and -4EBP1 systems[45]. It has been demonstrated that Egr is present in the promoter of KLF5 genes [43], and PPARγ activation may inhibit Egr activation and expression [46,47]. In this study we demonstrated that rosiglitazone inhibited Ang II-induced Egr DNA binding activity, which indicated that PPAR-γ agonist might regulate KLF5 expression by inhibiting Egr transcriptional activation. Taken together, our results indicated that AT1/PKCζ/ERK1/2/Egr signal pathways might implicate in the suppression of KLF5 expression and cell proliferation in VSMCs in response to rosilgitzone.

In summary, our findings provide evidence for the beneficial effects of the PPAR-γ agonist to counter-regulate KLF5 expression induced by Ang II. More importantly, anti-proliferation of PPAR-γ activation, by interfering with the Ang II /KLF5 signaling pathway, works in concert to protect against vascular remodeling.

## Supporting Information

S1 Fig Effects of siRNA on PPAR-γ, KLF5 and PKCζ expression. (A). After application of negative control siRNA (NC siRNA) or PPAR-γ siRNA for 48 hrs, VSMCs were subsequently treated with rosiglitazone (5 μM) for 24 hrs. (B). After application of negative control siRNA (NC siRNA) or KLF5 siRNA for 48 hrs, VSMCs were subsequently stimulated with Ang II (0.1 μM) for 24 hrs. (C). After application of negative control siRNA (NC siRNA) or PKCζ siRNA for 48 hrs, VSMCs were subsequently stimulated with Ang II (0.1 μM) for 24 hrs. For mRNA studies, results are showed as fold increase over control, and data are mean ± S.E.M. of 3 independent experiments. GAPDH was served as an internal control. For protein studies, results are showed as mean ± S.E.M. (bottom panel) of 3 independent experiments. Results are expressed as fold increase over control group. β-actin served as an internal control. (*P<0.05 vs. control).
(TIF)

S2 Fig The vascular morphology changes of the thoracic aorta in different groups. Cross-sections of thoracic aorta segments collected at the time of sacrifice were paraffin-embedded and stained with alizarin blue. Images are representative of 6 animals studied in each group. Scale bar = 100 μm.
(TIF)

S3 Fig Expression and activation of KLF5 in VSMCs. Cells were pretreated with or without rosiglitazone (Ros; 5μM) for 1 h and subsequently stimulated with Ang II (0.1 μM) for 24 h. Detection of KLF5 protein in nuclear extracts by western blot analysis. *TBP was served as an internal control. (*P<0.05 vs. control; #P<0.05 vs. Ang II.).
(TIF)
