# Supplementary figures and images for: Rosiglitzone Suppresses Angiotensin II-Induced Production of KLF5 and Cell Proliferation in Rat Vascular Smooth Muscle Cells
Source: PLoS One. 2015 Apr 14;10(4):e0123724. doi: 10.1371/journal.pone.0123724 (PMC4397085; doi:10.1371/journal.pone.0123724)

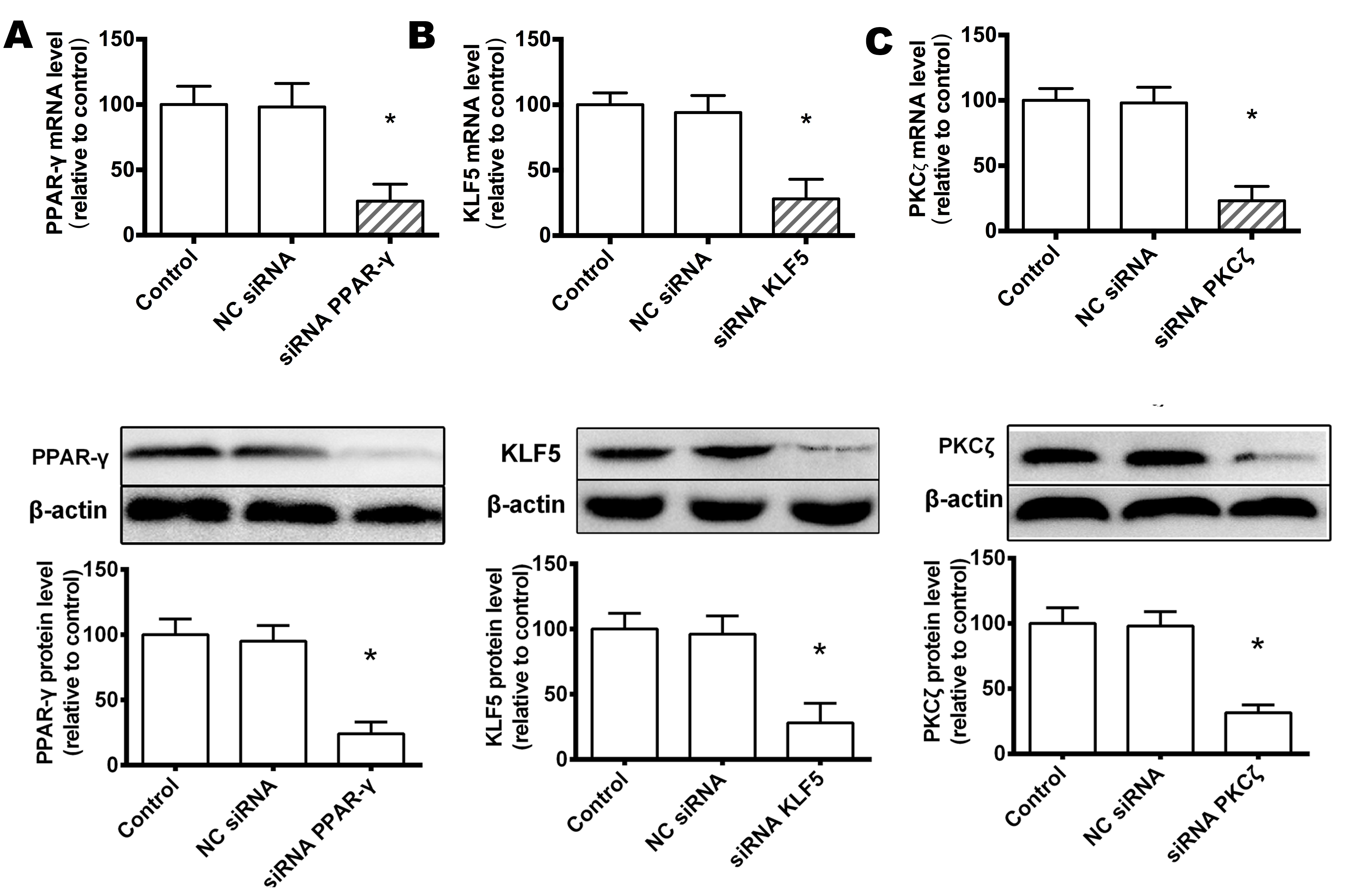

Supplement: S1 Fig — (A). After application of negative control siRNA (NC siRNA) or PPAR-γ siRNA for 48 hrs, VSMCs were subsequently treated with rosiglitazone (5 μM) for 24 hrs. (B). After application of negative control siRNA (NC siRNA) or KLF5 siRNA for 48 hrs, VSMCs were subsequently stimulated with Ang II (0.1 μM) for 24 hrs. (C). After application of negative control siRNA (NC siRNA) or PKCζ siRNA for 48 hrs, VSMCs were subsequently stimulated with Ang II (0.1 μM) for 24 hrs. For mRNA studies, results are showed as fold increase over control, and data are mean ± S.E.M. of 3 independent experiments. GAPDH was served as an internal control. For protein studies, results are showed as mean ± S.E.M. (bottom panel) of 3 independent experiments. Results are expressed as fold increase over control group. β-actin served as an internal control. (*P<0.05 vs. control). (TIF) [file pone.0123724.s001.tif]

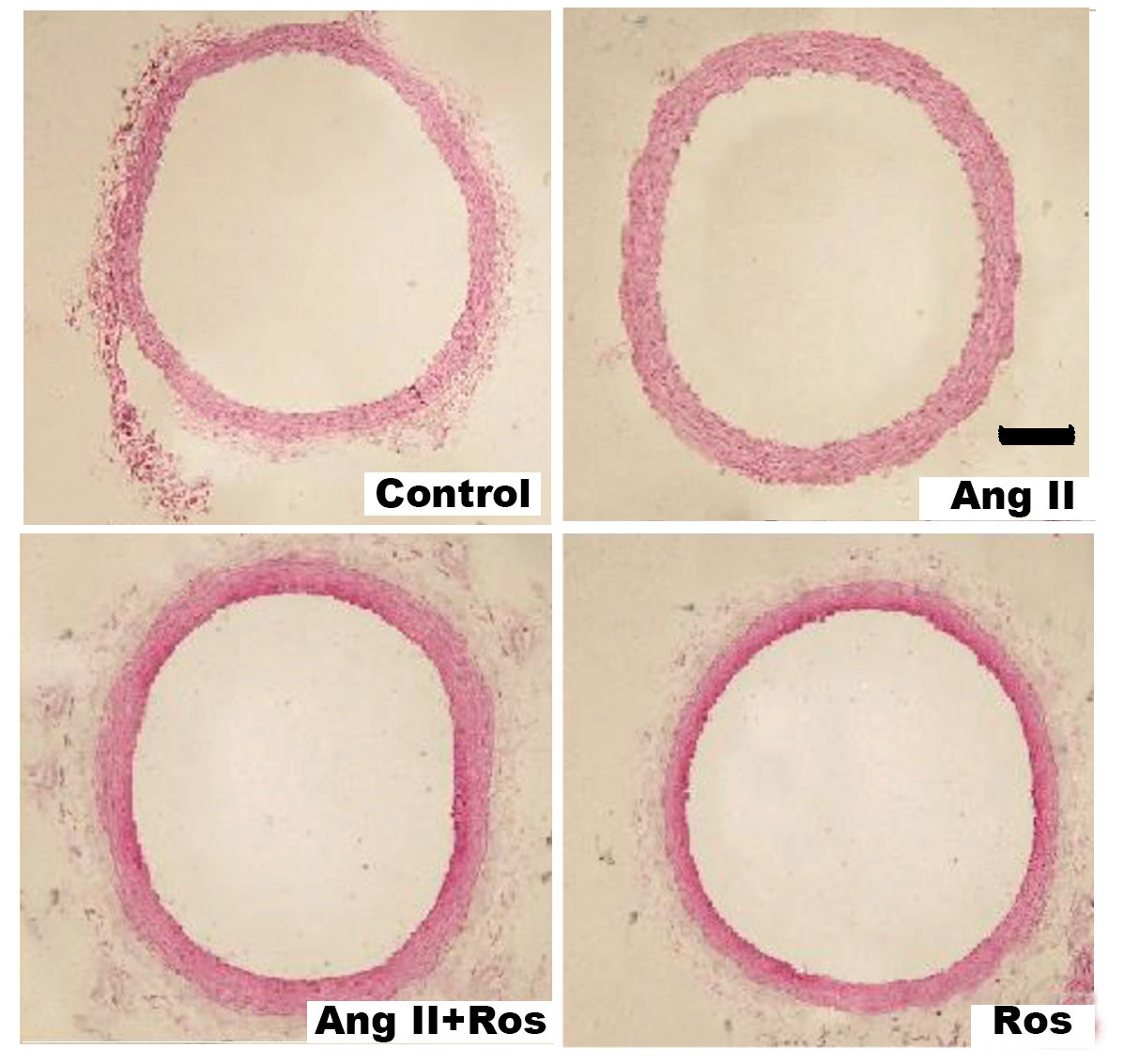

Supplement: S2 Fig — Cross-sections of thoracic aorta segments collected at the time of sacrifice were paraffin-embedded and stained with alizarin blue. Images are representative of 6 animals studied in each group. Scale bar = 100 μm. (TIF) [file pone.0123724.s002.tif]

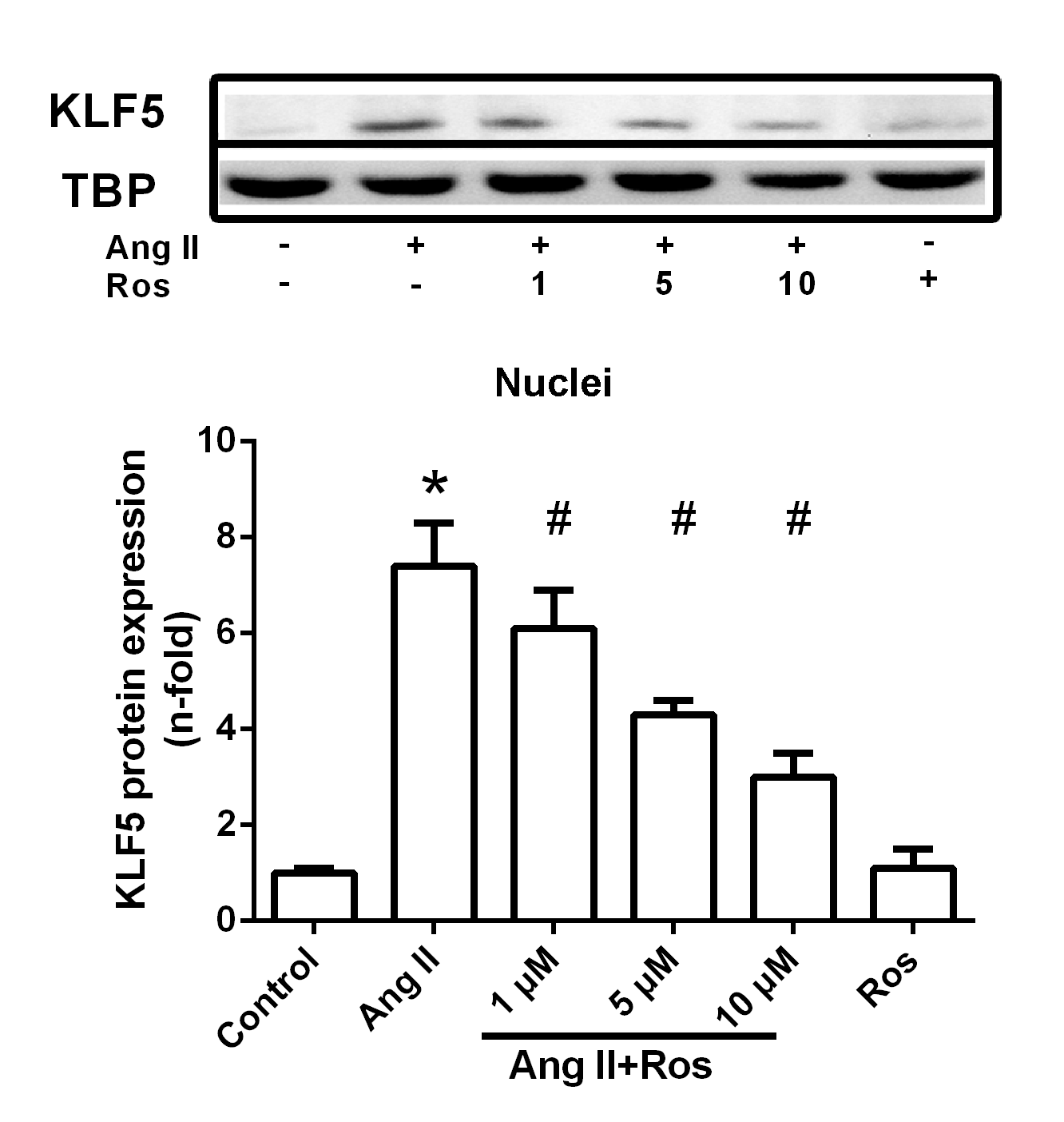

Supplement: S3 Fig — Cells were pretreated with or without rosiglitazone (Ros; 5μM) for 1 h and subsequently stimulated with Ang II (0.1 μM) for 24 h. Detection of KLF5 protein in nuclear extracts by western blot analysis. *TBP was served as an internal control. (*P<0.05 vs. control; #P<0.05 vs. Ang II.). (TIF) [file pone.0123724.s003.tif]
